# Electrocardiographic Diagnosis of Brugada Syndrome in a Patient with Right Bundle Branch Block

**DOI:** 10.19102/icrm.2022.13116

**Published:** 2022-11-15

**Authors:** Laszlo Littmann

**Affiliations:** ^1^Department of Internal Medicine, Atrium Health Carolinas Medical Center, Charlotte, NC, USA

In a recent issue of *The Journal of Innovations in Cardiac Rhythm Management*, Ali and Nilsson presented an interesting case of a patient with Brugada syndrome (BrS) who experienced an electrical storm triggered by coronavirus disease 2019 (COVID-19) and fever.^1^ Along with a similar publication,^2^ the case extends the spectrum of cardiovascular complications associated with COVID-19. For educational purposes, however, I feel one should point out that the illustrations in the article were not fully consistent with a diagnosis of BrS.

In patients with right bundle branch block (RBBB), wide and tall terminal positivity in lead V1 can be easily mistaken for an ST-segment elevation. A simple method for discriminating the QRS complexes from the ST segments is to use simultaneous leads with well-defined QRS complexes and to draw a vertical line at the end of the QRS complex. **Figure 1A** is such a labeled enlargement of the illustration by Ali and Nilsson demonstrating that there was no discernible ST-segment elevation in V1. Also, in patients with BrS, there is frequently an increase in ST-segment elevation before the onset of ventricular tachycardia or ventricular fibrillation, and the coupling interval of the first beat is almost always >340 ms.^3,4^ In the case presented, however, the onset of ventricular tachycardia was not preceded by ST-segment elevation, and the coupling interval of the first beat appeared to be extremely short at 220–240 ms **(Figure 1B)**.

In expert commentaries, Drs. Wu, Wilde, and Baranchuk et al. each discussed the difficulty of diagnosing BrS in patients with RBBB.^5^ In previous such cases, however, there were attempts to unearth the Brugada electrocardiogram (ECG) by demonstrating ST-segment elevation in V2 and V3 or in leads placed one interspace higher, by using sodium-channel blockade, or with special pacing maneuvers.^5^ In the case presented, no such corroborating evidence was provided.

Except for the fact that the described patient had previously proven BrS, the case demonstrated all the characteristics of idiopathic ventricular fibrillation associated with complete RBBB.^6–8^ In such patients, as in the case by Ali and Nilsson, the ECG demonstrates RBBB but no ST-segment elevation in the anterior chest leads, and the episodes of ventricular tachycardia and ventricular fibrillation start with short coupling intervals. In RBBB-associated idiopathic ventricular fibrillation, intravenous isoproterenol can also suppress an electrical storm.^8^ The interesting case by Ali and Nilsson can serve as an educational opportunity to highlight the complex relationship between RBBB, BrS, and idiopathic ventricular fibrillation.

## Figures and Tables

**Figure 1:**
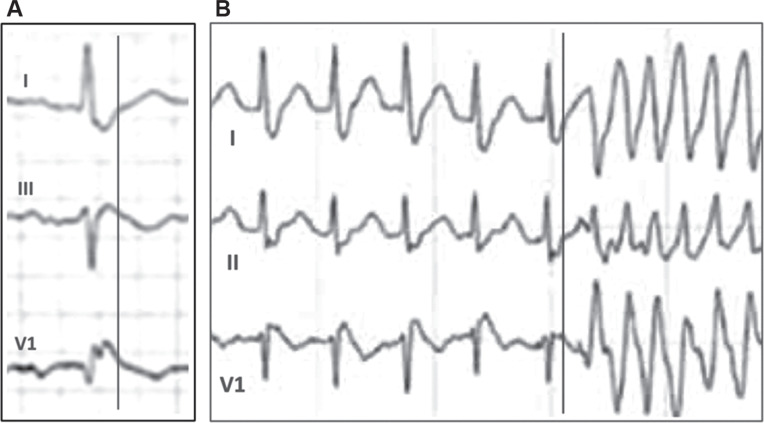
**A:** Enlargement of a segment of **Figure 1** from a publication by Ali and Nilsson.^1^ The vertical line represents the end of the QRS complex. Note the absence of ST-segment elevation in V1. **B:** Enlargement of a segment of **Figure 2** from the publication by Ali and Nilsson.^1^ Note the absence of ST-segment elevation before the onset of ventricular tachycardia, and note the extremely short coupling interval of approximately 220–240 ms of the first beat of the tachycardia.
